# Pathology in General Practice

**Published:** 1907-04-27

**Authors:** 


### PATHOLOGY IN GENERAL PRACTICE.

### The Demonstration of Tubercle Bacilli and other Acid-fast Bacilli.—(*Continued.*)

The diagnostic importance of the presence or absence of such bacilli in the sputum of respiratory cases is very great. Where tubercle exists in the lungs, and necrosis and breaking down of the tissues has taken place, then bacilli will be found quite easily; on the other hand, in obsolete tubercle of the apices, in some cases of miliary tubercle where breaking down has not commenced, and in tubercle of the pleura with no lung involvement, they will be absent. In any suspicious case three or four examinations on different days, preferably of the sputum coughed up in the early morning, should always be made, and if after this the result is negative a revision of the diagnosis is necessary. The mere presence of blood in a sputum, though suggestive, does not, of course, always mean that tubercle is present; such a condition may be caused by malignant disease of the lung, either primary or secondary, and also is common in cases of severe mitral stenosis with congestion of the lungs. An illustration or two will explain this more fully. A case with severe hæmoptysis and emaciation showed, on repeated examinations, no tubercle bacilli in the sputum; the diagnosis then veered round to "probably malignant disease," and this was eventually verified at the autopsy, the case being one of sarcoma of the supra-renal capsules with metastatic deposits which had undergone necrosis and breaking down, in the left lung. Again, a case exhibited blood in the sputum, which was very profuse and purulent, marked dullness all over the upper part of the right side of the chest, wasting, and eventually paraplegia, while no tubercle bacilli could ever be detected in the sputum. At the autopsy the condition was primary cancer of the lung with metastases in the spine. Every case of doubt must be carefully examined, and the test as a routine one must be constantly used.

### Leprosy.

Of other acid-fast bacilli, the leprosy bacillus comes next in importance. Cases of leprosy are much more common in England than is generally supposed, and as the bacteriological technique is exactly the same as for tubercle, the practitioner should be quite competent to diagnose such cases if he happens to meet with them in his practice. The bacillus resembles the tubercle bacillus very closely, both as regards its size, general appearance, and staining reactions; it has never, however, been cultivated, though many attempts to do so have from time to time been made. It is generally stated that the bacilli take up the basic anilin stains more readily than do those of tubercle, but this is doubtful; and in order to ensure success, carbol-fuchsin should be used. Again, some say the bacilli decolorise more easily, and so advise weaker acids (5 per cent.) to be used; but the usual 25 per cent. in our hands has always given quite satisfactory results. The position of the bacilli in the different parts of the body is interesting. They can generally be found in the nasal mucus; they are present in the tubercles in enormous numbers; they are comparatively scanty in the macular eruptions, and also in the nerves; they have also been found in the testicles, lymphatic glands, spleen, liver, and endothelium of the blood-vessels. Their enormous number in the skin lesions at once announces them as non-tubercular, and their marked tendency to become arranged in bundles is also useful in differentiating them. The best ways to obtain them for demonstration are the following: (1) With a platinum needle (looped) take some of the secretion from the nose, spread this out on a slide, and fix it in the usual manner over the spirit lamp. (2) Take an ivory pile clamp and squeeze one of the tubercles between its two limbs. Next open the tubercle with a small knife or needle and clear serum will exude. Take some of this, spread it on the slide, and fix it in the usual manner; then stain by the Ziehl-Nielsen method. Carbol-fuchsin is applied, the specimen is heated over the spirit-lamp till bubbles of steam rise, then the 25 per cent. sulphuric acid is poured on and off till the film becomes pale pink or almost white; next after washing well, counter-stain with methylene blue and mount in Canada balsam. The bacilli, just as in specimens prepared from tubercle, are red: the general tissue, pus cells, granulation tissue, and stroma, blue.

### Other Acid-fast Bacilli.

In addition to those of tubercle (under which we also include mammalian, avian, and fish tubercle) and leprosy, other acid-fast bacilli exist; and though these are of little importance of themselves from a pathological point of view, they may be mistaken for the bacilli of tubercle, and thus give rise to much confusion. The tests for distinguishing them are chiefly cultural, and cannot be gone into here, but the following brief description of them may prove useful:—

1. The Smegma bacillus, found in smegma: small bacillus, generally a little shorter than tubercle; difficult to stain; resists decoloration with strong mineral acids after being stained; grows with difficulty on culture media; may be confused with the tubercle bacillus in urinary cases, but may be distinguished by adding alcohol to the smear for a minute after the treatment with acid. This decolorises it, but does not affect the bacillus of tubercle. In suspected cases the urine should always be drawn off with a catheter.
2. Mœller's grass bacillus: strongly acid-fast. It is distinguished by the fact that it will grow easily and quickly on ordinary cultural media. From the fæces of the cow it might get into the milk, and so be mistaken for the bacillus of tubercle.
3. Mœller's mist bacillus (dung bacillus): acid-fast, similar characters to the grass bacillus.
4. Rabinawitch's bacillus of butter (butter bacillus): shorter and thicker than the bacillus of tubercle, acid-fast. Grows fairly rapidly on culture media.
5. Some of the streptothrix group are also acid-fast.

